# Contrast-induced encephalopathy and permanent neurological deficit following cerebral angiography: A case report and review of the literature

**DOI:** 10.3389/fncel.2022.1070357

**Published:** 2023-01-04

**Authors:** Yujing Zhang, Jiancheng Zhang, Shiying Yuan, Huaqing Shu

**Affiliations:** ^1^Department of Critical Care Medicine, Union Hospital, Tongji Medical College, Huazhong University of Science and Technology, Wuhan, China; ^2^Institute of Anesthesia and Critical Care Medicine, Union Hospital, Tongji Medical College, Huazhong University of Science and Technology, Wuhan, China

**Keywords:** cerebral angiography, contrast-induced encephalopathy, neurological deficit, prognosis, risk factor

## Abstract

Contrast-induced encephalopathy (CIE) is an uncommon complication associated with contrast exposure during angiographic procedures that is usually transient but occasionally leads to permanent complications or death. Due to the low incidence of CIE, there are still insufficient reports. This study was used to summarize the clinical features of CIE through a case report and systematic review. We summarized and reviewed 127 patients with CIE, and we found that the total incidence of CIE between men and women had no difference (49.61 and 50.39%, respectively), but the average age in female patients with CIE was older than that in male patients (62.19 and 58.77 years, respectively). Interestingly, the incidence of female patients with CIE in the poor prognosis group was significantly higher than that in the good prognosis group (62.50 and 36.51%, respectively), and the average age of these female patients in the poor prognosis group was younger than that in the good prognosis group (61.39 and 62.82 years, respectively). The contrast medium types were mainly nonionic (79.69 and 73.02%, respectively) and low-osmolar (54.69 and 71.43%, respectively) in both groups. Importantly, the total contrast media administrated in patients with poor prognoses was greater than that administrated in patients with good prognoses (198.07 and 188.60 ml, respectively). In addition, comorbidities in both groups included hypertension (55.91%), diabetes mellitus (20.47%), previous contrast history (15.75%), renal impairment (11.81%), and hyperlipidemia (3.15%). The percentage of patients with cerebral angiography was significantly higher in the poor prognosis group than that in the good prognosis group (37.50 and 9.52%, respectively), whereas the percentage of patients with coronary angiography in both groups had the opposite results (35.94 and 77.78%, respectively). In conclusion, CIE may not always have a benign outcome and can cause permanent deficits. Female gender, younger age, the higher dose of contrast medium, and the procedure of cerebral angiography may be related to the patient’s poor prognosis.

## Introduction

Contrast-induced encephalopathy (CIE) is an uncommon complication associated with intravenous or intra-arterial exposure to iodinated contrast media during angiographic procedures. The incidence of CIE ranges between 0.3 and 4.0% (de Bono, 1993; Potsi et al., 2012; Liu et al., 2020). Since the first description in 1970, the clinical features of CIE have included headache, memory loss, confusion, visual and speech impairment, seizures, hemiparesis, and even coma. The underlying mechanisms and causes of iodine-based CIE remain unclear. Studies suggest that this may be related to transient blood–brain barrier (BBB) breakdown and increased permeability, which may subsequently contribute to extravasation of contrast medium into the central nervous system, resulting in cerebral edema and altered neuronal excitability (Dangas et al., 2001; Babalova et al., 2021). In addition, some high concentrations of contrast media may cause the clumping of red blood cells and, consequently, occlusion of arterial branches, which may play a role in permanent neurological deficits (Cristaldi et al., 2021). Most patients with CIE have a good prognosis and resolve quickly within 1–2 days (Spina et al., 2017). A minority (approximately 15% of CIE) may develop permanent neurological deficits or fatal cerebral edema (Hamra et al., 2017; Donepudi and Trottier, 2018; Zhao et al., 2019). However, the development of an evidence-based consensus on CIE has been hindered by the low incidence of CIE. Although the current literature on CIE is extensive, only case reports in the literature describe CIE and further analyses on the risk factors of CIE prognosis have been rarely performed. Here, we provided a case report as a reference and summarized existing reports about CIE, aiming to explore pathogenesis, risk factors, diagnosis, treatment strategy, and future exploration direction of the disease.

## Case report

A 51-year-old woman was admitted to our hospital with a suspected intracranial aneurysm. The patient had a history of hypertension. On admission, a physical examination showed no signs of neurological deficits. Cerebral angiography was urgently performed through the right femoral approach. The procedure lasted for 60 min. A total of 50 ml iodixanol (Jiangsu Hengrui Pharmaceutical Co., Ltd., China), an iso-osmolar non-ionic dimeric hydrophilic contrast medium, was injected. Notably, 1% lignocaine was administered for local anesthesia prior to cerebral angiography. This was the patient’s first exposure to a contrast medium, and no obvious aneurysm or vascular malformation was found.

**FIGURE 1:**
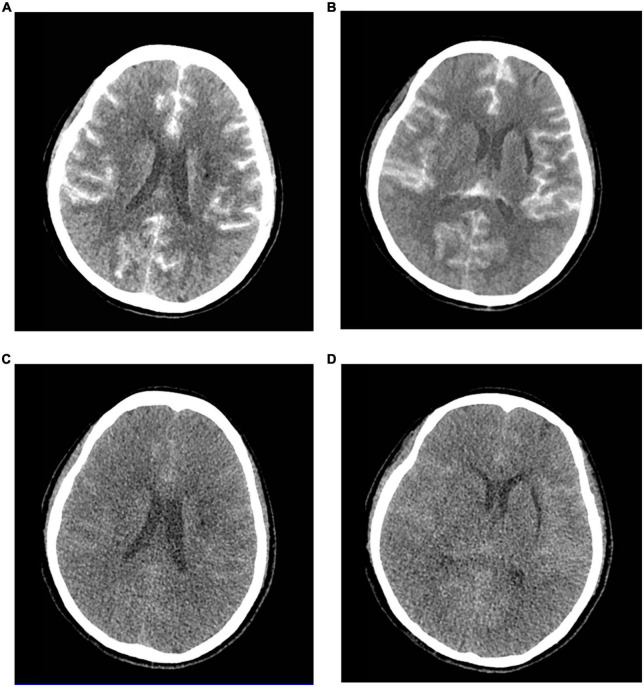
Contrast-induced encephalopathy in brain computed tomography (CT) scans. **(A,B)** Emergency brain CT 5 h after the procedure showed diffuse contrast enhancement in the brain parenchyma and subarachnoid space with mild global brain edema and the softening foci in the left basal ganglia-insular area. **(C,D)** Brain CT 2 days after the procedure indicated diffuse enhancement disappeared, and the brain parenchyma was diffusely swollen and the lateral ventricle was slightly more compressed.

Approximately 5 h after surgical completion, the patient developed a decrease in the upper and lower extremity motor strength, and the pupils were symmetric and reactive. The left muscle strength was grade 2, and the right muscle strength was grade 3. An emergency brain computed tomography (CT) scan was requested and revealed the diffuse contrast enhancement in brain sulci, fissures, cisterns, third ventricle, fourth ventricle, and subarachnoid space with mild global brain edema, and softening foci in the left basal ganglia-insular area (Figures 1A,B). On the second day, the patient’s clinical symptoms further deteriorated and the upper and lower muscle strength was grade 0 with positive pathological signs. Brain CT was reviewed and showed diffuse enhancement disappeared, but the brain parenchyma was diffusely swollen and the lateral ventricles were slightly more compressed than that in the previous scan (Figures 1C,D). A diagnosis of CIE was suspected given the worsening of the clinical manifestations and symptoms compatible with higher functional impairment following the administration of the contrast medium.

The patient routinely received fluids to accelerate the excretion of contrast medium, 1,000 mg of intravenous methylprednisolone once daily for 2 days to mitigate inflammation, 250 ml of mannitol every 8 h to dehydrate and reduce intracranial pressure, 10 mg of nimodipine once daily to prevent vasospasm, 120 mg of sodium valproate once daily to prevent epilepsy, as well as strengthen nutrition to improve clinical symptoms. Furthermore, lumbar cistern drainage was performed to reduce intracranial pressure, and cerebrospinal fluid (CSF) was clear with increased white blood cell count and glucose level and decreased chloride level. In the following hours, the patient experienced further deterioration in mental status and fell into a coma with respiratory insufficiency. Therefore, the patient was transferred to the intensive care unit (ICU) where she underwent tracheal intubation with ventilator-assisted breathing, dehydration, anti-epileptic therapy, body temperature and blood pressure control, and close neurological observation.

**FIGURE 2:**
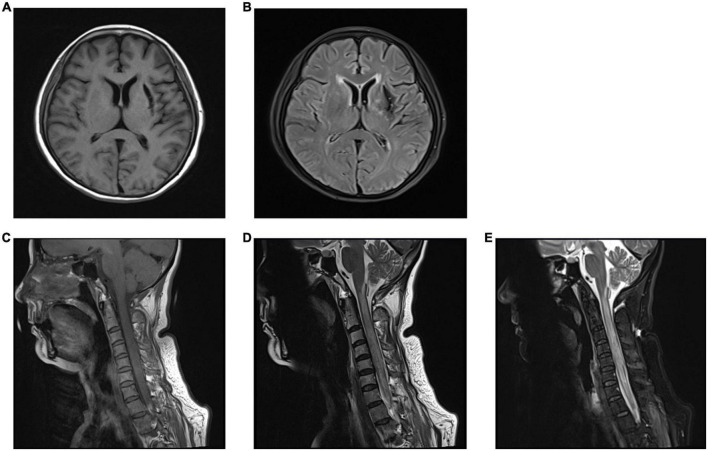
The brain and cervical spine magnetic resonance imaging (MRI) performed at 2 weeks. **(A,B)** Brain MRI showed the left basal ganglia-insular softening foci in T1 **(A)** and T2 **(B)** weighted image. **(C–E)** Cervical spine MRI showed diffuse hyperintense signal in the cervical cord in T2-weighted image **(D)** and fluid-attenuated inversion recovery (FLAIR) images **(E)**, and normal findings were observed in T1-weighted image **(C)**.

On the second day after being admitted to the ICU, the patient regained consciousness, but her motor deficit was unchanged. A neurological examination showed muscle weakness in the upper and lower limbs and sensory loss below the T2 sensory level, which may be related to spinal cord edema. Considering that the patient was temporarily unable to remove the tracheal tube, a tracheotomy was performed 4 days later. A magnetic resonance imaging (MRI) performed at 2 weeks revealed a diffuse hyperintense signal on FLAIR sequences in the cervical cord, which may be consistent with the patient’s motor deficits and sensory disturbances, as well as a softening foci formation in the left basal ganglia-insular area (Figure 2). Dramatically, the patient suffered from a lung infection during hospitalization and was eventually discharged from the neurosurgery ward to another hospital for hyperbaric oxygen therapy after 20 days. A telephone follow-up after 2 months revealed that the patient’s persistent neurological deficits had not improved.

## Literature review

**FIGURE 3:**
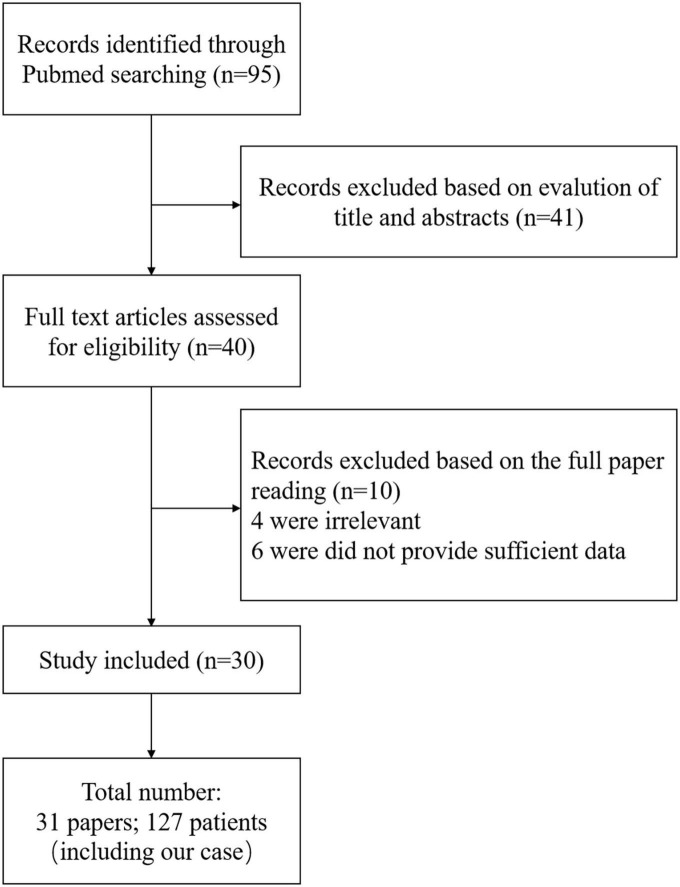
Screening process.

In searching for the keywords “Contrast-induced encephalopathy” and “Angiography” in PubMed, 95 relevant articles were found. A total of 54 papers were selected after screening abstracts and titles. After reading through the whole paper, the reviews, editorial, and duplicate cases were excluded, and 40 papers were left. However, four of them were excluded because it was defined as allergic reactions, vasospasm, and posterior reversible encephalopathy syndrome, and complete data were not available in the other six papers. Finally, we accurately summarized 30 papers (Leong and Fanning, 2012; Yan and Ramanathan, 2013; Kocabay et al., 2014; Nagamine et al., 2014; Hamra et al., 2017; Park et al., 2017; Spina et al., 2017; Dattani et al., 2018; Heemelaar et al., 2018; Hirata et al., 2018; Kahyaoğlu et al., 2018; Tong et al., 2018; Renault and Rouchet, 2019; Riahi et al., 2019; Şimşek et al., 2019; Zhao et al., 2019, 2021; Fernando et al., 2020; Harada et al., 2020; Lei et al., 2020; Liu et al., 2020; Andone et al., 2021; Cristaldi et al., 2021; García-Pérez et al., 2021; Kamimura et al., 2021; Li et al., 2021; Vigano et al., 2021; Yao et al., 2021; Zhang et al., 2021; Rashid et al., 2022). A total of 127 patients were enrolled. Figure 3 shows the screening process. Table 1 shows the basic information of 31 studies (including our case).

**TABLE 1:** Basic information of all patients.

Study	Number	Gender/Age (year)	Procedure	Risk factor	Previous angio- graphy	Contrast medium	Contrast medium class	Volume (ml)	Presentation	Neuroimaging	Symptom duration	Complete resolution
Andone et al., 2021	1	M/70	Diagnostic coronary angiography	HT, DM	No	Ioversol	Non-ionic, monomer, low osmolar	100	Headache, behavioral changes and aggressive tendencies	CT: a mild hyper-density in the frontal lobes; MRI: normal	72 h	Yes
Dattani et al., 2018	2	M/76	Coronary angiography	HT, DM	No	Iohexol	Non-ionic, monomer, low osmolar	120	Confused and aggressive, expressing verbal profanities	CT: normal	9 days	Yes
	3	M/65	Diagnostic coronary angiography	Previous CIE, HT, Dyslipidaemia, Smoking.	Yes	Iopromide	Non-ionic, monomer, low osmolar	110	Global aphasia, bilateral limb weakness	CT and MRI: normal;	24 h	Yes
	4	M/49	Coronary angiography	–	–	Iopromide	Non-ionic, monomer, low osmolar	205	Confusion, decreased GCS	–	12 h	Yes
	5	M/32	Coronary angiography	–	–	Iopromide	Non-ionic, monomer, low osmolar	100	Cortical blindness	CT: normal	24 h	Yes
	6	F/39	Coronary angiography	–	No	Iopamidol	Non-ionic,monomer, low osmolar	100	Cortical blindness	CT: normal	1 h	Yes
	7	M/74	Coronary angiography + aortogram	HT, Obse	No	Iomeprol	Non-ionic, monomer, low osmolar	320	Cortical blindness	CT: normal	24 h	Yes
	8	M/73	Coronary angiography + PCI	Sleep apnea, Hypoventilation, Obese	–	None reported	N/A	240	Cortical blindness	CT: normal	7 h	Yes
	9	M/53	Coronary angiography	–	–	Ioversol	Non-ionic, monomer, low osmolar	100	Cortical blindness, catatonia	–	12 h	Yes
	10	M/45	Coronary angiography + PCI	HT	No	Ioversol	Non-ionic, monomer, low osmolar	167	Cortical blindness	–	24 h	Yes
	11	M/59	Coronary angiography + PCI	HT	No	Ioversol	Non-ionic, monomer, low osmolar	220	Cortical blindness	–	12 h	Yes
	12	M/68	Coronary angiography + PCI	HT	No	Ioversol	Non-ionic, monomer, low osmolar	262	Homonymous hemianopia	–	15 min	Yes
	13	M/55	Coronary angiography	–	Yes	Diatrizoate	Ionic, monomer, high osmolar	280	Cortical blindness	CT: both occipital lobes enhancement	24 h	Yes
	14	M/61	Coronary angiography	–	Yes	Diatrizoate	Ionic, monomer, high osmolar	145	Cortical blindness	CT: normal	36 h	Yes
	15	M/44	Coronary angiography	–	No	Diatrizoate	Ionic, monomer, high osmolar	50	Cortical blindness	No brain CT	12 h	Yes
Kamimura et al., 2021	16	F/70	Diagnostic erebral angiography	Not reported	No	Iopamidol	Non-ionic, monomer, low osmolar	43	Confusion and generalized tonic-clonic seizures	CT: high-density signaling in the cortex; MRI: high signal intensity in temporal lobe	24 h	Yes
	17	M/71	Cerebral angiography	Not reported	No	Iohexol	Non-ionic, monomer, low osmolar	46	Cortical blindness, confusion, and Ophthalomoplegia	No brain CT	10 days	N/A
	18	F/68	Cerebral angiography	Not reported	No	Iohexol	Non-ionic, monomer, low osmolar	24	Cortical blindness, confusion, and amnesia	No brain CT	6 days	N/A
	19	F/71	Cerebral angiography	HT, Transient ischemic attack	No	Iopromide	Non-ionic, monomer, low osmolar	25	Confusion, disorientation, and hemiparesis	CT: edema, right diffuse cortical hyperattenuation	24 h	Yes
Fernando et al., 2020	20	F/52	Coronary angiography	HT	N/A	Iopromide	Non-ionic, monomer, low osmolar	130	Left sided hemiparesis, disorientation, and decreased GCS	CT: cortical and subarachnoid hyper-densities	5day	Yes
Cristaldi et al., 2021	21	F/54	Cerebral angiography	HT, Cerebral ischemia	N/A	Iobitridol	Non-ionic, monomer, low osmolar	Not reported	Severe right hemiparesis and complete aphasia	CT: edema, abnormal subarachnoid contrast enhancement zone	>6 months	No
	22	M/10	Angiocardio- graphy	Fallot’s tetralogy	Not stated	Urografin 76%	Ionic, monomer, high osmolar	76	Apnoea and cardiac arrest	No CT brain (autopsy showed brain edema)	During	Death
	23	F/7	Aortography	HT	No	Renografin 76%	Ionic, monomer, high osmolar	340	Seizures	CT: contrast enhancement of cortex, basal ganglia and thalamus	During	Death
	24	M/68	Bypass graft angiography	HT, DM	Yes	Iohexol	Non-ionic, monomer, low osmolar	180	Confusion, amnesia, aphasia, cortical blindness	CT: contrast enhancement of occipital lobes, temporal lobes, thalami	During	No
	25	F/73	Coronary angiography	HT	No	Iohexol	Non-ionic, monomer, low osmolar	1150	Seizures, gait instability, postural tremor, dysphasia	CT: abnormal signal in bilateral frontal and occipital	During	No
	26	F/62	Cerebral angiography	None reported	No	Not specified	Non-ionic	297	Confusion, cortical blindness	CT: abnormal signal in bilateral occipital, basal ganglia, frontal	During	No
	27	M/41	Cerebral angiography	None reported	Yes	Not specified	Non-ionic	225	Bilateral visual loss, agitation	CT: right parietal abnormal signal	3 h	No
	28	F/54	Cerebral angiography	None reported	Yes	Not specified	Non-ionic	62	Cortical blindness with only light perception	MRI: bilateral occipital	1 months	Yes
	29	M/46	Cerebral angiography	None reported	Yes	Not specified	Non-ionic	225	Right homonymous hemianopia	CT and MRI: normal	1 months	Yes
	30	M/47	Cerebral angiography	None reported	No	Not specified	Non-ionic	384	Right homonymous hemianopia	CT: normal	7 days	Yes
	31	F/67	Coronary angiography	Angina, DM, HT	–	Iodixanol	Non-ionic, dimer, iso-osmolal	Not reported	Gradually GCS 3	N/A	During	Yes
	32	F/71	Cerebral angiography	HT, hyperlipidemia, Angina	–	Iopamidol	Non-ionic, monomer, low osmolar	110	Dizziness, nausea, vomiting; then respiratory distress, deep coma, GCS 3	CT: cerebral edema	56 days	Death
	33	F/51	Carotid artery angioplasty	HT, DM, Coronary artery disease	–	Iohexol	Non-ionic, monomer, low osmolar	Not reported	Seizures, unconsciousness	N/A	During	Death
Spina et al., 2017	34	F/44	Diagnostic coronary angiography + PCI	End-stage kidney disease, HT, DM	–	Iohexol	Non-ionic, monomer, low osmolar	190	Left-sided weakness, seizure activity	CT: contrast enhancement of right cerebral hemisphere	72 h	Yes
	35	M/69	Diagnostic coronary angiography + PCI	HT, DM	–	Not reported	N/A	N/A	Aphasia, left-sided hemiparesis	CT: contrast enhancement of right cerebral hemisphere	12 h	Yes
	36	F/63	Diagnostic coronary angiography	HT, DM	–	Iopramide	Non-ionic, low osmolar	250	Cortical blindness	CT: contrast enhancement of occipital lobes; MRI: normal	72 h	Yes
	37	F/60	Diagnostic coronary angiography	HT	–	Not reported	N/A	N/A	Abrupt decrease in GCS score to 6/15	CT: cerebral edema confined to the right cerebral hemisphere; MRI: normal	10 days	Yes
	38	F/76	Diagnostic coronary angiography	HT, DM	–	Ioversol	Non-ionic, monomer, low osmolar	125	Aphasia, cortical blindness, right sided weakness	MRI: hyperintensity in frontoparietal regions	48 h	Yes
	39	F/69	Diagnostic coronary angiography + PCI	CKD, DM, Previous contrast reaction	Yes	Iodixanol	Non–ionic, dimer, iso-osmolal	320	Partial seizure, homonymous hemianopia, hemisensory loss, hemiparesis	CT: cerebral edema	24 h	Yes
	40	M/64	Diagnostic coronary angiography + PCI	HT, DM	–	Iopromide	Non-ionic, monomer, low osmolar	160	Confusion, irritability, limb paralysis, aphasia	CT: hyperdensity of sagittal sinus	28 h	Yes
	41	M/68	Diagnostic coronary angiography + PCI	HT	–	Iopromide	Non-ionic, monomer, low osmolar	250	Left lower extremity weakness and sensory loss	CT: contrast enhancement in sagittal sinus and occipital lobe	12 h	Yes
	42	M/47	Diagnostic coronary angiography + PCI	None reported	–	Iopromide	Non-ionic, monomer, low osmolar	150	Confusion, agitation, nausea, headache	CT: contrast enhancement in right occipital lobe	8 h	Yes
	43	M/70	Diagnostic coronary angiography + PCI	DM	–	Iopromide	Non-ionic, monomer, low osmolar	120	Confusion; nausea	CT: contrast enhancement in occipital lobe	12 h	Yes
	44	F/76	PCI + carotid artery stenting	CKD, DM	–	Iodixanol	Non-ionic, dimer, iso-osmolal	200	Stupor, aphasia, hemiparesis	CT: hyperdensity of cerebral sulci and subarachnoid spaces	48 h	Yes
	45	F/39	Diagnostic coronary angiography	–	–	Iopamidol	Non-ionic, monomer, low osmolar	80	Cortical blindness	CT and vertebral angiogram: normal	1 h	Yes
	46	F/70	Diagnostic coronary angiography + PCI	–	–	None reported	N/A	N/A	Left-sided hemiparesis, conjugate gaze deviation to the right	CT: hyperdensity of cerebral sulci and right frontal lobe	72 h	Yes
	47	F/70	Diagnostic coronary angiography + PCI	–	–	Not specified	Ionic	1500	Myoclonus	CT: hyperdensity of cerebral sulci	<1 h	Yes
	48	F/52	Diagnostic coronary angiography	–	–	Iomeprol	Non-ionic, monomer, low osmolar	150	Cortical blindness	CT: contrast enhancement of occipital lobes	5 h	Yes
	49	F/70	Diagnostic coronary angiography	DM, HT	–	Iobitridol	Non-ionic, monomer, low osmolar	75	Cortical blindness	CT: contrast enhancement of occipital lobes	72 h	Yes
	50	M/56	Diagnostic coronary angiography	–	–	Iopromide	Non-ionic, monomer, low osmolar	135	Confusion, dysarthria, cortical blindness	CT: contrast enhancement of right occipital lobe	24 h	Yes
	51	F/82	Diagnostic coronary angiography + PCI	CKD, HT	–	Iomeprol	Non-ionic, monomer, low osmolar	500	Aphasia, right-sided hemiparesis	CT: hyperdensities filling the sulci of both cerebral hemispheres	40 h	Yes
	52	M/82	Diagnostic coronary angiography	CKD, DM, HT	–	Iopromide	Non-ionic, monomer, low osmolar	150	Right-sided hemiparesis, aphasia	CT: Left hemisphere cerebral edema and extravascular local contrast media	6 h	Yes
	53	M/63	Diagnostic coronary angiography + Aortogram	–	–	Iopremol	Non-ionic, monomers	450	Amnesia, numbness, right upper extremity numbness	CT: contrast enhancement of right occipital lobe	12 h	Yes
	54	F/63	Diagnostic coronary angiography	DM, HT	–	Iopromide	Non-ionic, monomer, low osmolar	160	Cortical blindness, right homonymous hemianopia	CT and MRI: contrast enhancement of occipital lobes	48 h	Yes
	55	F/52	Diagnostic coronary angiography + PCI	HT	Yes	Ioversol	Non-ionic, monomer, low osmolar	280	Cortical blindness	CT: contrast enhancement of occipital lobes	36 h	Yes
	56	M/55	Diagnostic coronary angiography	–	–	Iomeprol	Non-ionic, monomer, low osmolar	280	Cortical blindness	CT: contrast enhancement of occipital lobes	5 days	Yes
	57	M/58	Diagnostic coronary angiography	HT	–	Ioglaxate	Ionic, dimer, low osmolar	260	Cortical blindness	CT: normal	32 h	Yes
	58	M/64	Diagnostic coronary angiography	–	–	Ioglaxate	Ionic, dimer, low osmolar	400	Cortical blindness	N/A	30 h	Yes
	59	M/49	Diagnostic coronary angiography	CKD, HT	–	Diatrizoate	Ionic, monomer, high osmolar	610	Seizures, encephalopathy	CT: contrast enhancement of left frontal gyri	4 h	Yes
	60	M/62	Diagnostic coronary angiography + PCI	CKD, HT	–	Iopamidol	Non-ionic, monomer, low osmolar	170	Headache, confusion, cortical blindness	CT: contrast enhancement of cerebellum, thalamus	12 h	Yes
	61	M/62	Diagnostic coronary angiography	HT	–	Iopamidol	Non-ionic, monomer, low osmolar	270	Cortical blindness, loss of coordination right arm	CT: contrast enhancement of occipital lobes	72 h	Yes
	62	F/57	Diagnostic coronary angiography	HT, Previous contrast reaction	Yes	Ioglaxate	Ionic, dimer, low osmolar	200	Cortical blindness	CT: mild attenuation in occipital poles	48 h	Yes
	63	F/52	Diagnostic coronary angiography + aortogram	HT	–	Diatrizoate	Ionic, monomer, high osmolar	100	Cortical blindness	N/A	18 h	Yes
Hamra et al., 2017	64	M/62	Coronary angioplasty	HT	–	Iohexol	Non-ionic, monomer, low osmolar	200	Right-sided homonymous hemianopia	CT: contrast enhancement of the venous sinuses and cerebral arteries	48 h	Yes
Vigano et al., 2021	65	F/56	Cerebral angiography	Migraine, Renal colic, Smoking, Previous heroin abuse	–	Iomeprol	Non-ionic, monomer, low osmolar	70	Global aphasia and right hemiplegia	CT: left cerebral edema	10 days	Yes
	66	F/74	Abdominal aorta and renal artery angiography + angioplasty	Renal impairment; HT	No	Diatrizoate	Ionic, monomer, high osmolar	250	Cortical blindness, left hemiparesis	CT: bilateral occipital and basal ganglia alterations	4–5 days	Yes
	67	M/64	Carotid artery and aorta angiography	HT	No	Iothalamate meglumine	Ionic, monomer, high osmolar	12	Cortical blindness, fluent aphasia	CT: left temporo-parieto-occipital alterations	3 days	N/A
	68	F/71	Spinal angiography	–	No	Ioxaglate	Ionic dimer low osmolar	360	Right-sided visual neglect and Wernicke’s aphasia	CT: bilateral occipital and left parietal lobe alterations	4 days	Yes
	69	M/82	Carotid artery angiography + stenting	HT	Yes	Ioxaglate	Ionic, dimer, low osmolar	50	Confusion, left hemiparesis, neglect	CT: right frontoparietal cortical enhancement and edema	2 days	Yes
	70	M/72	Carotid artery angiography + coiling anterior aneurysm	–	No	Iopamidol	Non-ionic, monomer, low osmolar	260	Right hemiparesis and motor aphasia	CT: enhancement throughout the left cerebral cortex and left basal ganglia, diffuse swelling of the left cerebral hemisphere	7 days	Yes
	71	M/80	Carotid and coronary angiography + stenting	HT	No	Iohexol	Non-ionic, monomer, low osmolar	250	Right hemiparesis	CT: left frontoparietal-occipital cortical enhancement	2 days	Yes
	72	M/51	Carotid artery angiography + right internal carotid artery	HT	No	Iopromide	Non-ionic, monomer, low osmolar	300	Gerstmann’s left visual field deficit, hemiparesis, right gaze deviation	CT: cortical enhancement and edema in the right cerebral hemisphere	2 days	Yes
	73	M/69	Coronary angiography + PCI	–	–	Iohexol	Non-ionic, monomer, low osmolar	100	Stupor, disorientation, left hemiplegia	CT: hyperdense lesion in the right frontoparietal region, parietal lobe and basal ganglia	6 h	Yes
	74	F/73	Aortic angiography + thoracic aortic aneurysm repair	Chronic kidney disease; HT	Yes	Iodixanol	Non-ionic, dimer, iso-osmolal	248	Seizure and left-sided hemiplegia	CT: hyperdensity of the right cortex, subarachnoid space and basal ganglia	7 days	N/A
	75	F/67	Cerebral angiography	HT	Yes	N/A	N/A	N/A	Right-sided hemiparesis and aphasia	CT: cortical edema of the left cerebral hemisphere and contrast medium leakage to the subarachnoid space	24 h	N/A
Liu et al., 2020	76	F/84	Coronary angiography	HT, Paroxysmal atrial fibrillation, Chronic bronchitis	–	Iopromide	Non-ionic, monomer, low osmolar	20	Lost consciousness, and exhibited left limb hemiplegia with muscle strength level 0 and eyes staring to the right, seizure	CT: high-density regions in the subarachnoid space	2 months	Yes
	77	F/57	Diagnostic coronary angiography + PCI	CKD, HT, DM	–	Iodixanol	Non-ionic, dimer, iso-osmolal	130	Tonic–clonic seizures	CT: right parenchymal edema	72 h	Yes
	78	M/6	Diagnostic coronary angiography	HT, DM	–	Iohexol	Non-ionic, monomer, low osmolar	120	Confused, aggressive, expressing verbal profanities	CT: normal	9 days	Yes
	79	M/62	Diagnostic coronary angiographyc + PCI	HT	–	Iohexol	Non-ionic, monomer, low osmolar	300	Right-sided homonymous hemianopia	CT: slight enhancement of the venous sinuses	48 h	Yes
	80	M/49	Diagnostic coronary angiography	–	–	Iopromide	Non-ionic, monomer, low osmolar	205	Confusion, decrease in level of consciousness	CT and MRI: normal	12 h	Yes
	81	M/69	Diagnostic coronary angiography + PCI	–	–	Iohexol	Non-ionic, monomer, low osmolar	100	Confusion, headache, vomiting, left hemiplegia,	CT: focal hyperdense lesions	6 h	Yes
	82	M/74	Diagnostic coronary angiography + aortogram		–	Iomeprol	Non-ionic, monomer, low osmolar	320	Cortical blindness	CT: normal	24 h	Yes
Riahi et al., 2019	83	F/71	Coronary angiography	HT, DM	–	Iodixanol	Non-ionic, dimer, iso-osmolal	80	Aphasia, GCS score to 7/15	CT: normal	24 h	Yes
Rashid et al., 2022	84	F/76	Coronary angiography	DM, HT, Hyperlipidemia, Coronary artery disease	–	N/A	N/A	N/A	Confusion and aggressive behavior	CT and MRI: normal	16 days	Yes
Zhang et al., 2021	85	M/42	Coronary angiography	–	–	Iopromide	Non-ionic, monomer, low osmolar	200	Severe headache, cortical blindness and neuropsychiatric symptom	CT: normal	5 days	Yes
Park et al., 2017	86	F/58	Cerebral angiography	HT, Hypothyroidism, Peripheral artery occlusive disease, Depressive disorder	–	Iodixanol	Non-ionic, dimer, iso-osmolal	220	Tonic-clonic seizure, left hemiparesis involving face, arm and leg(grade 3/5), sensory loss, and left-sided neglect with drowsy mentality	CT:sulcal obliteration of right cerebral hemisphere; MRI: gyral swelling and hyperintensity in the right cerebral hemisphere	6 days	Yes
Kocabay et al., 2014	87	F/58	Diagnostic coronary angiography + PCI	HT, Hyperlipidemia	–	Iopromide	Non-ionic, monomer, low osmolar	220	Bilateral oculomotor ophthalmoplegia	N/A	>30 days	No
	88	M/68	Diagnostic coronary angiography + PCI	HT	–	Iopromide	Non-ionic, monomer, low osmolar	250	Monoplegia	N/A	12 h	Yes
	89	M/68	Diagnostic coronary angiography + PCI	HT, DM	–	Iopromide	Non-ionic, monomer, low osmolar	180	Unilateral oculomotor monoplegia	N/A	1 h	Yes
	90	M/70	Diagnostic coronary angiography + PCI	HT	–	Iopromide	Non-ionic, monomer, low osmolar	130	Cerebellar dysfunction	N/A	14 h	Yes
Kahyaoğlu et al., 2018	91	M/66	Lower extremity angiography	–	–	Iohexol	Non-ionic, monomer, low osmolar	Not reported	Confusion and cortical blindness, seizure	CT: normal	24 h	Yes
Zhao et al., 2019	92	F/71	Digital subtraction angiography	HT, Hyperlipemia, Angina	–	Iopamidol	Non-ionic, monomer, low osmolar	110	Headache, dizziness, nausea and vomiting, deep coma	CT: cerebral edema	56 days	Death
Li et al., 2021	93	F/77	Digital subtraction angiography	HT, Coronary heart disease	Yes	Visipaque	Non-ionic	200	Right hemiplegia, aphasia, and epilepsy	CT: hyperdensity in the left subarachnoid space	6 days	Yes
Zhao et al., 2021	94	F/50	Cerebral angiography	–	–	Iopromide	Non-ionic, monomer, low osmolar	6	Disturbance of consciousness, seizures, frequent blinking, and stiffness	CT: normal; MRI: swelling of the left cerebral cortex	10 days	Yes
Heemelaar et al., 2018	95	F/67	Coronary angiography	DM, HT, Adenocarcinoma of the left breast	–	Iso-osmolar iodinated contrast	Iso-osmolar	100	Acute-onset coma and respiratory insufficiency	CT: bilateral cerebral edema	23 days	Yes
García-Pérez et al., 2021	96	F/61	Diagnostic digital subtraction angiography	Migraine	–	Iodixanol	Non-ionic, dimer, iso-osmolar	70	Confused and drowsy, agitated and vomiting	MRI: lesions in the cerebellar hemispheres and parieto-occipital lobes;	36–48 h	Yes
	97	M/22	Diagnostic digital subtraction angiography	Left parietooccipital arteriovenous malformation	–	None reported	N/A	None reported	Disoriented, bilateral amaurosis and presented amnesia	CT and MRI: normal	48–72 h	Yes
Harada et al., 2020	98	F/72	Coronary angioplasty	HT, Hyperlipidemia	–	Iodinated contrast	None reported	210	Left hemiparesis, left sensory and visual hemineglect, and right gaze preference	CT: mild cerebral edema	2 days	Yes
Renault and Rouchet, 2019	99	M/49	Renal artery angiography	Chronic renal failure	–	Iohexol	Non-ionic, monomer, low osmolar		Cortical blindness, global amnesia disappeared	MRI: normal	6 days	Yes
Hirata et al., 2018	100	M/75	Coronary angioplasty	DM	–	None reported	N/A	None reported	No neurological symptoms were observed because the patient was intubated	CT: high-density areas in the cortex, putamen, caudate nucleus and subarachnoid space of the right cerebral hemisphere	12 days	Yes
Yao et al., 2021	101	M/68	Contrast-enhanced chest CT examination	Rheumatoid arthritis	–	Iso-osmolar iodinated contrast	Iso-osmolar	70	Lost consciousness and experienced cardiorespiratory arrest	CT: abnormal cortical contrast enhancement and cerebral sulci hyperdensity	>17days	Yes
Leong and Fanning, 2012	102	F/50	Cerebral angiography	HT	No	Iopramide	Non-ionic, low osmolar	220	Right hemisyndrome	CT: edema in the left cerebral hemisphere	During	No
	103	F/59	Aortic arch angiography	Renal impairment; HT	Yes	Diatrizoate	Ionic, monomer, high osmolar	150	Cortical blindness; headache, myoclonus or seizure	CT: bilateral parieto-occipital contrast enhancement	3 days	Yes
	104	F/53	Carotid artery angiography	Not reported	No	Diatrizoate meglumine	Ionic, monomer, high osmolar	60	Partial motor seizure	CT: right temporo-parietal contrast enhancement	24 h	Yes
	105	M/70	Arch, Carotid & Vertebral arteries angiography	None reported	No	Diatrizoate	Ionic, monomer, high osmolar	72	Cortical blindness	CT: bilateral Occipital hyperdensity	2 days	Yes
	106	F/74	Abdominal aorta angiography	HT, Renal impairment	No	Iopamidol	Non-ionic, monomer, low osmolar	415	Visuospatial disorder	CT: bilateral parieto-occipital hyperdensity	4 days	Yes
	107	F/74	Diagnostic cerebral angiography	HT	No	Iohexol	Non-ionic, monomer, low osmolar	None reported	Complete bilateral blindness; confusion	CT: left parieto-occipital hyperdensity; MRI: left occipita hyperdensity	24 h	Yes
	108	F/45	Diagnostic cerebral angiography	HT	No	Iohexol	Non-ionic, monomer, low osmolar	None reported	Complete bilateral blindness; confusion	CT: normal; MRI: bilateral occipital hyperdensity	7 days	Yes
	109	F/73	Diagnostic cerebral angiography	HT	No	Iohexol	Non-ionic, monomer, low osmolar	None reported	Cortical blindness	CT: normal; MRI: bilateral occipita hyperdensity	5 days	Yes
	110	F/70	Coronary artery angiography	HT	Yes	None reported	N/A	1500	Seizure	CT: hyperdensity of right frontal	24 h	Yes
	111	M/56	Coronary artery	None reported	Yes	Iohexol 350	Non-ionic, monomer, low osmolar	220	Bilateral cortical blindness	CT: high-density areas in the bilateral occipital and frontal lobes	4 days	Yes
Nagamine et al., 2014	112	F/58	Cerebral angiography	Unknown	N/A	Iohexol	Non-ionic, monomer, low osmolar	–	Agraphia, right hemiparesis	N/A	>20 days	No
Tong et al., 2018	113	F/64	Diagnostic cerebral angiography	HT	No	ioversol	Non-ionic, monomer, low osmolar	300	Lateral blindness	MRI: normal	6 days	Yes
	114	M/53	Diagnostic cerebral angiography	HT	Yes	Omnipaque	Non-ionic, monomer, high osmolar	155	Lateral blindness	CT: bilateral brain edema on frontal and occipital lobe	5 days	Yes
	115	F/61	Diagnostic cerebral angiography	HT	–	Omnipaque	Non-ionic, monomer, high osmolar	10	Lateral blindness	MRI: right occipital cerebellar infarction	3 months	Yes
	116	M/58	Carotid artery + vertebral artery angiography	–	Yes	Iopromide	Non-ionic, monomer, low osmolar	350	Cortical blindness	CT: cortical hyperdensity and vasogenic edema	10 days	Yes
	117	M/57	Vertebral artery angiography	–	Yes	Omnipaque	Non-ionic, monomer, high osmolar	20	Bilateral cortical blindness	CT: normal; MRI: abnormal bilateral parieto-occipital lobes	24 h	Yes
Lei et al., 2020	118	M/76	Coronary angiography	HT, DM, Transient ischemic attacks	Yes	Iodixanol	Non-ionic, dimer, iso-osmolar	150	Epileptic seizures	N/A	6 months	Yes
Yan and Ramanathan, 2013	119	M/63	Cerebral angiogram	HT, DM, Rheumatoid arthritis, End-stage renal disease	–	Iodixano	Non-ionic, dimer, iso-osmolar	910	Left-sided blindness and ophthalmoplegia	CT: bilateral subarachnoid hyper-attenuation over the cerebral sulci, and diffuse cerebral edema	72 h	Yes
	120	F/58	Cerebral arteriogram	–	–	Iodixanol	Non-ionic, dimer, iso-osmolar	193	Left hemiparesis	CT: hyperdensity of right frontoparietal lobe	4 days	Yes
	121	F/61	Cerebral arteriogram	–	–	Iodixanol	Non-ionic, dimer, iso-osmolar	212	Left hemiparesis	CT: hyperdensity of right frontoparietal lobe	3 days	Yes
	122	F/58	Transfemoral cerebral angiography	–	–	Ioversol	Non-ionic, monomer, low osmolar	N/A	Global aphasia, right sided hemiparesis	CT: abnormal enhancement in left cerebral cortex and thalamus	3 days	Yes
	123	F/63	Right middle cerebral angiography	–	–	Iopromide	Non-ionic, monomer, low osmolar	150	N/A	CT: diffuse subarachnoid hyperdensity	2 days	Yes
	124	M/16	Angiography of the circle of Willis	–	–	Iopromide	Non-ionic, monomer, low osmolar	50	Cortical blindness	CT: occipital lobe hyperdensity	24 h	Yes
	125	F/69	Coronary angioplasty	–	–	Iopamidol 370	Non-ionic, monomer, low osmolar	260	Seizure, right hemiparesis.	CT: left cerebral cortex, left basal ganglia hyperdensity	18 h	Yes
Şimşek et al., 2019	126	M/68	Coronary angiography	HT, DM, Coronary artery disease, Severe renal impairment	No	Iohexol	Non-ionic, monomer, low osmolar	230	seizure	CT: hyperdense fields at the vertex and at the right frontal lobe	60 h	Yes
Our case	127	F/51	Cerebral angiography	HT	No	Iodixanol	Non-ionic, dimer, iso-osmolal	50	Dyskinesia, coma	CT:the diffuse hyperdensity in brain sulci, fissures, cisterns, third ventricle, fourth ventricle and subarachnoid space, and global brain edema	>3 months	No

From the search results, we found that the total incidence of CIE between women and men has no difference. A total of 63/127 (49.61%) patients were women and 64/127 (50.39%) patients were men, and the average age in women was older than that in men (62.19 and 58.77 years, respectively). More importantly, we classified the statistical results according to prognosis, and patients who recovered less than or equal to 48 h were included in the good prognosis group and the remaining patients were included in the poor prognosis group. Eventually, 63 patients were included in the good prognosis group and 64 patients were included in the poor prognosis group, and the results are shown in Table 2. We found that the incidence of female patients with CIE in the poor prognosis group was significantly higher than that in the good prognosis group (62.50 and 36.51%, respectively), and the average age of these female patients in the poor prognosis group was younger than that in the good prognosis group (61.39 and 62.82 years, respectively). Furthermore, the poor prognosis group had a wider age range, ranging from 6 to 84 years.

**TABLE 2:** The relationship between prognosis with variable.

	≤2 days (*n* = 63)	>2 days (*n* = 64)
**Gender**		
Female (%)	23 (36.51)	40 (62.50)
Male (%)	40 (63.49)	24 (37.50)
Age (year)	62.82 ± 1.41	61.39 ± 1.45
**Contrast types**		
Non-ionic (%)	46 (73.02)	51 (79.69)
Ionic (%)	12 (19.05)	6 (9.38)
Low-osmolar (%)	45 (71.43)	35 (54.69)
High-osmolar (%)	8 (12.70)	7 (10.94)
Iso-osmolal (%)	4 (6.35)	11 (17.19)
Contrast doses (ml)	188.60 ± 13.99	198.07 ± 14.23
Non-ionic (ml)	193.39 ± 15.59	199.19 ± 14.73
Ionic (ml)	167.70 ± 37.91	235.20 ± 54.49
Low-osmolar (ml)	195.10 ± 16.18	207.83 ± 19.85
High-osmolar (ml)	117.83 ± 35.31	194.20 ± 45.73
Iso-osmolal (ml)	167.50 ± 58.79	152.56 ± 23.40
**Comorbidities**		
Hypertension (%)	32 (50.79)	39 (60.93)
Diabetes mellitus (%)	10 (15.78)	16 (25.00)
History of angiography (%)	9 (14.29) + 1	11 (17.19)
Renal impairment (%)	6 (9.52)	9 (14.06)
Dyslipidaemia (%)	2 (3.17)	3 (4.69)
**Angiography types**		
Coronary angiography (%)	49 (77.78)	23 (35.94)
Cerebral angiography (%)	6 (9.52)	24 (37.50)
Carotid and vertebral angiography (%)	6 (9.52)	4 (6.25)
Abnormal CT or MRI (%)	40 (63.49)	44 (68.75)

In interventional procedures, the contrast types included non-ionic and ionic, or low-osmolar, high-osmolar, and iso-osmolar in our present study, and we found that both groups were mainly non-ionic (79.69 and 73.02%, respectively) and low-osmolar (54.69 and 71.43%, respectively). Importantly, the total contrast media administrated and the non-ionic, ionic, low-osmolar, or high-osmolar contrast media administrated in patients with poor prognosis were greater than that administrated in patients with good prognosis (198.07 and 188.60 ml, 199.19 and 193.39 ml, 235.20 and 167.70 ml, 207.83 and 195.10 ml, and 194.20 and 117.83 ml, respectively), whereas the iso-osmolar contrast media administrated was lower in patients with poor prognosis compared to patients with good prognosis (152.56 and 167.50, respectively).

The comorbidities in the present study mainly included hypertension (55.91%), diabetes mellitus (20.47%), previous contrast history (15.75%), renal impairment (11.81%), and hyperlipidemia (3.15%). Although there was no significant difference in comorbidities between the two groups, the percentages of hypertension, diabetes mellitus, previous contrast history, renal impairment, and hyperlipidemia in the poor prognosis group were higher than those in the good prognosis group (60.93 and 50.79%, 25.00 and 15.78%, 17.19 and 4.29%, 14.06 and 9.52%, and 4.69 and 3.17%, respectively). The angiography types, mainly coronary angiography (56.69%), cerebral angiography (23.62%), and carotid and vertebral angiography (7.87%), in both groups, were also analyzed. We found that the percentage of patients with cerebral angiography in the poor prognosis group was significantly higher than that in the good prognosis group (37.50 and 9.52%, respectively), whereas the percentage of patients with coronary angiography in both groups had the opposite results (35.94 and 77.78%, respectively). Moreover, brain CT or MRI abnormalities were found in most patients in both groups (68.83 and 62.00%, respectively).

## Discussion

Contrast-induced encephalopathy is a rare and reversible complication that can cause neurotoxicity with a favorable prognosis and resolves within 24–48 h in most cases. Based on previous studies (Yan and Ramanathan, 2013; Spina et al., 2017; Cristaldi et al., 2021), the renal elimination of contrast medium, the regression of cerebral edema, and the recovery of BBB function were assumed to play an important role in the pathophysiology of neurological recovery. Here, we described a case of permanent neurological deficit after cerebral angiography and provided a summary and analysis of a series of CIE cases to explore the probable reasons for permanent neurological deficit. Given that most patients resolved completely within 48 h, we performed a prognostic analysis using 48 h as the node. We found that the total incidence of CIE between female patients and male patients had no difference, but female patients were more likely to have a poor prognosis. In addition, the average age of patients with poor prognoses was younger than that of patients with good prognoses. Surprisingly, no reports are currently available on risk factors associated with prognosis in patients with CIE. Only two reports were found to analyze the relationship between the incidence of CIE and gender or age, and the conclusions of the two reports were inconsistent. One report found that the adverse drug reaction incidence of iodinated contrast medium (e.g., CIE) seemed to be associated with gender, with a significantly higher incidence in female patients than in male patients, and it was also associated with age, with a lower occurrence in older (>44 years) patients compared to younger patients (Jiang et al., 2021). The other report summarized 9 CIE cases in 2013 and proposed that male gender and advanced age are the greatest risk factors for developing CIE. These two reports just provide a reference for us, and further research and a more in-depth analysis are necessary.

Studies showed a correlation between contrast medium dose and CIE (Yu and Dangas, 2011; Vigano et al., 2021), and whether the more contrast medium used is related to the poor prognosis of patients has not been directly reported. Although our study showed that the patients with poor prognosis used more contrast medium among different types of contrast media, including the non-ionic, ionic, low-osmolar, and high-osmolar contrast media, as well as the total contrast media used, the results are not absolute. Because in our reported case and 4 other summarized cases, the patient presented with permanent neurological deficits (more than 10 days) after administrating only a low quantity of contrast medium (no more than 50 ml) for angiography. Among these cases, the contrast medium types included non-ionic, low-osmolar, high- osmolar, and iso-osmolar, suggesting that severe neurotoxic symptoms may occur in response to low doses and different types of contrast agents. A previous study has shown that a 49-year-old man developed CIE and completely resolved within 4 h after receiving 610 ml diatrizoate (an ionic high-osmolar contrast medium) for diagnostic coronary angiography (Muruve and Steinman, 1996), indicating that high-dose contrast media do not cause permanent neurological dysfunction. Therefore, we speculated that, in addition to volume, the poor prognosis is generally related to the route and number of administrated, type of contrast medium, and individual patient characteristics.

Previous research has shown that demographic risk factors for CIE are chronic hypertension, diabetes mellitus, renal insufficiency, and previous reactions to contrast media (Yu and Dangas, 2011; Zhao et al., 2019; Cristaldi et al., 2021). Our study showed that the majority of patients (55.91%) had hypertension, 20.47% had diabetes mellitus, 15.75% had a contrast history, and 11.81% had renal insufficiency. Although there is no statistical difference between the poor prognosis group and the good prognosis group, these risk factors have a higher proportion in patients with poor prognosis, suggesting they may be related to worse prognosis, and further research is needed by increasing the sample size.

For the types of angiographic procedures, the present study showed that the proportion of patients with cerebral angiography was significantly higher in the poor-prognosis group than in the good-prognosis group, whereas patients with coronary angiography had the opposite results. Whether the cerebral angiography procedure itself is more likely to aggravate the prognosis than coronary angiography is unclear. This study demonstrated that 170 ml is recommended as the maximum threshold level of toxicity for coronary angiography procedure, and a smaller volume of contrast media may damage the BBB during selective intracranial injection (Kocabay et al., 2014), suggesting that cerebral angiography may be more likely to damage the BBB than coronary angiography. Furthermore, it is unclear whether procedure-related factors and patient-related factors are involved.

The diagnosis of CIE often requires the exclusion of cerebrovascular accidents such as cerebral hemorrhage and cerebral infarction. Neuroimaging plays an important role in distinguishing CIE from other neurological pathologies such as thromboembolism and hemorrhage following angiography. Our research showed that the most common abnormalities on brain CT included cortical or subcortical contrast enhancement, cerebral edema, focal hyperdense lesions, and hyper-density in the cerebral sulci. MRI abnormalities included hyperintensity on T2, FLAIR, and DWI. This study has suggested that CSF examination is also useful to rule out subarachnoid hemorrhage through the absence of xanthochromia or red blood cells (Shahan et al., 2021). The simultaneous detection of high concentrations of iodinated contrast medium in CSF and serum supports contrast medium extravasation rather than hemorrhage. In addition, the exclusion of contrast allergy or allergic-like reactions is also essential for the diagnosis of CIE. A recent study showed that allergic-like or allergic reactions caused by contrast media are rare, which can be severe or even life-threatening (Fusco et al., 2022). It is important to obtain a history of immediate or delayed reactions to a specific contrast medium, which may contribute to predicting the risk for future reactions. Clinical manifestations such as throat tightness, facial edema, and bronchospasm are helpful in distinguishing.

For the treatment of CIE, most patients with CIE have a good prognosis and a rapid recovery. Therefore, supportive care and observation are generally considered sufficient. Based on the literature summarized in the present study, it is recommended that appropriate hydration, steroids, and mannitol can be given immediately after surgery, and benzodiazepines can be used for epileptic seizures.

## Conclusion

A contrast-induced encephalopathy is a form of neurotoxicity caused by contrast media that is usually transient but occasionally leads to permanent complications or death. We summarized a series of cases and found that the female gender, younger age, higher contrast medium dose, and cerebral angiography procedure were associated with poor prognosis in patients with CIE. However, the contrast medium types were not associated with the prognosis. In addition, there was no statistical difference between the poor prognosis group and the good prognosis group; hypertension, diabetes mellitus, renal insufficiency, and previous reactions to contrast media were also important risk factors for CIE. Our case and literature review highlight that CIE may not always have a benign outcome and has the potential to cause permanent neurological dysfunction, even with low-dose contrast media. We should not be overlooked, especially following procedures that use contrast medium.

## Author contributions

YZ wrote the manuscript. JZ analyzed the data. HS and SY critically revised and edited the manuscript. All authors discussed the content and read and approved the final version.
